# Flipping Off and On the Redox Switch in the Microcirculation

**DOI:** 10.1146/annurev-physiol-031522-021457

**Published:** 2023-02-10

**Authors:** Máté Katona, Mark T. Gladwin, Adam C. Straub

**Affiliations:** 1 Pittsburgh Heart, Lung, and Blood Vascular Medicine Institute, University of Pittsburgh, Pittsburgh, Pennsylvania, USA; 2 Pulmonary, Allergy, and Critical Care Medicine, Department of Medicine, University of Pittsburgh School of Medicine, Pittsburgh, Pennsylvania, USA; 3 Current affiliation: University of Maryland School of Medicine, Baltimore, Maryland, USA; 4 Department of Pharmacology and Chemical Biology, University of Pittsburgh School of Medicine, Pittsburgh, Pennsylvania, USA; 5 Center for Microvascular Research, Department of Medicine, University of Pittsburgh School of Medicine, Pittsburgh, Pennsylvania, USA

**Keywords:** endothelial, smooth muscle, red blood cell, redox, calcium, microcirculation

## Abstract

Resistance arteries and arterioles evolved as specialized blood vessels serving two important functions: (*a*) regulating peripheral vascular resistance and blood pressure and (*b*) matching oxygen and nutrient delivery to metabolic demands of organs. These functions require control of vessel lumen cross-sectional area (vascular tone) via coordinated vascular cell responses governed by precise spatial-temporal communication between intracellular signaling pathways. Herein, we provide a contemporary overview of the significant roles that redox switches play in calcium signaling for orchestrated endothelial, smooth muscle, and red blood cell control of arterial vascular tone. Three interrelated themes are the focus: (*a*) smooth muscle to endothelial communication for vasoconstriction, (*b*) endothelial to smooth muscle cell cross talk for vasodilation, and (*c*) oxygen and red blood cell in-terregulation of vascular tone and blood flow. We intend for this thematic framework to highlight gaps in our current knowledge and potentially spark interest for cross-disciplinary studies moving forward.

## INTRODUCTION

The microcirculation encompasses a collection of small-diameter blood vessels: resistance arteries (50–200 μm), arterioles (20–50 μm), meta-arterioles branching to precapillary sphincters (10–20 μm), capillaries (<10 μm), and venules (10–250 μm). Resistance arteries and arterioles, the focus of this review, have two essential functions: (*a*) to govern peripheral vascular resistance and blood pressure and (*b*) modulate blood flow to capillaries for nutrient and oxygen (O_2_) delivery and waste product removal (e.g., CO_2_). Anatomically, resistance arteries and arterioles contain a single layer of endothelial cells (ECs) lining the vessel lumen and perpendicularly oriented contractile vascular smooth muscle cells (VSMCs) in single or multiple layers. ECs and VSMCs sandwich a thin internal elastic lamina matrix, which provides elasticity and adhesivity for cells. While resistance arteries and arterioles are surrounded by a thick VSMC wall, capillaries are encircled by thin multifunctional mural cells called pericytes, which have some contractile properties within certain organ systems (1). Maintenance of homeostatic blood pressure and blood flow in resistance arteries and arterioles requires constant modulation of vessel lumen cross-sectional area or vascular tone. Endocrine, paracrine, and autocrine signals continually trigger vascular tone adaptations to changing conditions within the organism. This constant on-demand control of vascular tone relies on direct cross-communication between ECs and VSMCs via input signals from red blood cells (RBCs), sympathetic nerves, and other circulating cells. Notably, resistance arteries and arterioles have evolved unique anatomical structures called myoendothelial junctions (MEJs) that protrude through the internal elastic lamina with diameters that are typically ~0.5 μm. MEJs directly connect ECs and VSMCs, serving as essential communication hubs for rapid electrical signaling and exchange of second messengers that regulate vascular tone in a highly organized and spatial-temporal fashion (2, 3). The density of MEJs inversely correlates with the luminal size of resistance arteries and arterioles (3). A central regulator of vascular tone is cytosolic Ca^2+^ ([Ca^2+^]_cyto_), which is essential for EC and VSMC function, albeit in a contrasting manner. In VSMCs, elevated [Ca^2+^]_cyto_ causes vasoconstriction, and Ca^2+^ sparks generally cause vasodilation. By contrast, increases in EC [Ca^2+^]_cyto_ elicit vasodilation. Vascular Ca^2+^ homeostasis is tightly controlled by redox signaling and redox switches that enable fine-tuning of [Ca^2+^]_cyto_ signaling pathways for organ-specific vascular tone regulation. Redox switches arise from natural or imbalanced oxidative posttranslational modifications to proteins with metal centers or thiol bases, affecting pathways for folding, localization, quality control, and degradation of proteins. Importantly, redox switches represent rapid, low-energy mechanisms of sensing and regulation of cellular homeostasis or defense. Over the past decade, we and others have come to appreciate that vascular cells rely on precision redox signaling to provide a stable and efficient microenvironment for the right reactive oxygen species (ROS) and reactive nitrogen species (RNS), i.e., the right location, the right timing, the right level, and the right target (4). In this review, we aim to highlight important contributions made to the field by summarizing key contributions of redox switches to Ca^2+^ signaling and vascular tone control.

## REACTIVE NITROGEN SPECIES/REACTIVE OXYGEN SPECIES: ORIGIN, REGULATION, AND TARGETS

RNS are continually produced in ECs and VSMCs, signaling at volumes determined by physiological and pathophysiological conditions. Endogenous RNS mostly originate from nitric oxide (NO) produced by neuronal, inducible, and endothelial nitric oxide synthase (eNOS) activity (5). NO is a lipophilic, membrane-permeable molecule that participates in near diffusion-limited reactions regulating physiological functions, including intestinal motility, platelet aggregation, proliferation, apoptosis, and vascular tone (6). NO is a free radical owing to its unpaired electron, making it highly reactive with other radicals and transition metals like ferrous iron–containing hemoproteins. NO exerts biological actions primarily through three cellular signaling cascades: (*a*) activation of its receptor soluble guanylyl cyclase (sGC), catalyzing the generation of cyclic guanosine 3′,5′-cyclic monophosphate (cGMP) for stimulating protein kinase G (PKG); (*b*) radical–radical reactions with superoxide, nitrogen dioxide, and lipid radicals, yielding RNS; and (*c*) posttranslational modification of cysteine (Cys) residues via indirect *S*-nitrosation, causing protein functional changes. In subsequent sections, we highlight cGMP-dependent and cGMP-independent signaling mechanisms in resistance arteries and arterioles.

ROS, such as hydrogen peroxide (H_2_O_2_), superoxide (O_2_^·−^), and hydroxyl radical (^·^OH), are major oxidants with specific signaling actions (7). Two major sources of ROS are mitochondria and oxidases. Mitochondria produce ROS via electron leak in the electron transport chain (complexes I, II, and III) and α-keto acid dehydrogenase complexes (8). The membrane-bound NADPH oxidases (NOXs) also produce ROS (i.e.,O_2_^·−^ and H_2_O_2_) when electrons are transferred between NADPH and O_2_ (9). Endogenous and exogenous ROS are also produced as by-products of extracellular xanthine oxidoreductase activity (10). In disease states, eNOS can also generate ROS (O_2_^·−^) when its reductase and oxidase domains become uncoupled (11). Iron-sulfur cluster oxidation, DNA damage, and peroxynitrite formation are consequences of O_2_^·−^ reactions (12), and protein thiols and transition metals, such as iron and copper, are targeted by H_2_O_2_ (13).

Cells have evolved enzymes for controlling levels of O_2_^·−^ and H_2_O_2_. There are three superoxide dismutases (SOD1–3) for catalyzing dismutation of O_2_^·−^ levels to H_2_O_2_: cytosolic expressed SOD1, mitochondria-localized SOD2, and extracellular-restricted SOD3 (14). Likewise, numerous degradation systems have evolved to control H_2_O_2_. Catalase is central to catabolizing cellular H_2_O_2_ to O_2_ and water in a two-step reaction (15). Glutathione (GSH) can act as a redox buffer against oxidative and electrophilic stress (16).When GSH reacts with H_2_O_2_,glutathione disulfide (GSSG) is formed, and GSH levels are restored by NADH-dependent GSH reductase (17). GSH can serve as a cosubstrate for glutathione peroxidase (GPx) degradation of H_2_O_2_ into H_2_O (18). Peroxiredoxins (Prx) are highly expressed antioxidant enzymes that can also degrade H_2_O_2_ and alkyl peroxide in collaboration with thioredoxins (Trx) (19).

Protein redox modifications can be categorized into two groups: irreversible and reversible. Irreversible modifications leading to protein carbonyls, nitrotyrosine, and sulfonic acid generally cause protein aggregation and degradation (20). Reversible modification is involved in signaling and regulation of protein function, occurring on cysteine residues of proteins: *S*-sulfenation, *S-*nitrosation, disulfide bond formation, *S*-glutathionylation, and methionine sulfoxide (21). Redox cycling of transition metals (e.g., iron and copper) also occurs (22). In subsequent sections, we summarize how resistance arteries and arterioles rely on redox balance and Ca^2+^ signaling to control vascular tone.

## SMOOTH MUSCLE TO ENDOTHELIAL COMMUNICATION AND CONTROL OF VASOCONSTRICTION

### Phase 1: Redox Regulation of Calcium Signaling in Vascular Smooth Muscle Cells and Contraction

Vascular tone is largely modulated through (*a*) G protein–coupled receptor (GPCR) activation, (*b*) shear stress on ECs, and (*c*) intraluminal pressure. These stimuli trigger extracellular Ca^2+^ entry or store [sarcoplasmic or endoplasmic reticulum (SR/ER) and lysosomes] operated Ca^2+^ release, increasing VSMC and EC [Ca^2+^]_cyto_ for calmodulin-dependent activation of myosin regulatory light chain (RLC20) phosphorylation and actin-myosin crossbridge formation (23). Simultaneously, the activation of RhoA-dependent kinase inhibits myosin light chain phosphatase from dephosphorylating RLC20, thereby sustaining VSMC contraction.

In VSMCs, Ca^2+^ influx occurs via plasma membrane voltage-gated and nonvoltage-gated pathways in response to mechanical, electrical, or hormonal stimuli. Electrophysiology studies identified two voltage-gated Ca^2+^ currents (VGCCs) in isolated VSMCs: transient (T-type) and long-lasting (L-type) (24). T-type Ca^2+^ channel currents (TTCCs) become activated at a lower, hyperpolarized membrane potential and decreased intraluminal pressure than L-type Ca^2+^ channel currents (LTCCs); however, they become inactivated faster than LTCCs (25). LTCCs consist of five different subunits (α_1_, α_2_, δ, β, and γ) (26). The α_1_ subunit of LTCCs is encoded by Ca_V_1 (Ca_V_1.1–3) genes and contain voltage-sensing, pore, and gating domains. The α_1_C subunit, encoded by Ca_V_1.2, is recognized as the primary voltage-gated Ca^2+^ influx pathway and is key to myogenic and receptor-mediated (α_1_-adrenergic and angiotensin 2) vasoconstriction in VSMCs (27). Inactivation of LTCC channel activity occurs by calmodulin binding to an isoleucine-glutamate domain, preventing cytotoxicity from Ca^2+^ overload (28). Protein kinases (PKA, PKC, and PKG) also affect LTCC activity in VSMCs, exemplified by NO-cGMP-PKG signaling for reduced LTCC current and VSMC vasodilatation (29). The Ca^2+^ activated K^+^ (BK) channel and voltage-gated K^+^ channels regulate LTCC activity by stimulating membrane potential change, as shown by LTCC deactivation with membrane depolarization (30). BK channel activity is increased by NO-cGMP-PKG signaling and through *S*-nitrosation. Redox sensing by LTCCs also regulates its activity, as shown by increased Ca^2+^ influx in ischemic human heart following H_2_O_2_ oxidization of Ca_V_1.2 Cys543 and subsequent glutathionylation, effects that were reversible by thiol reducing agents (dithiothreitol or GSH) (31).

**Figure 1:**
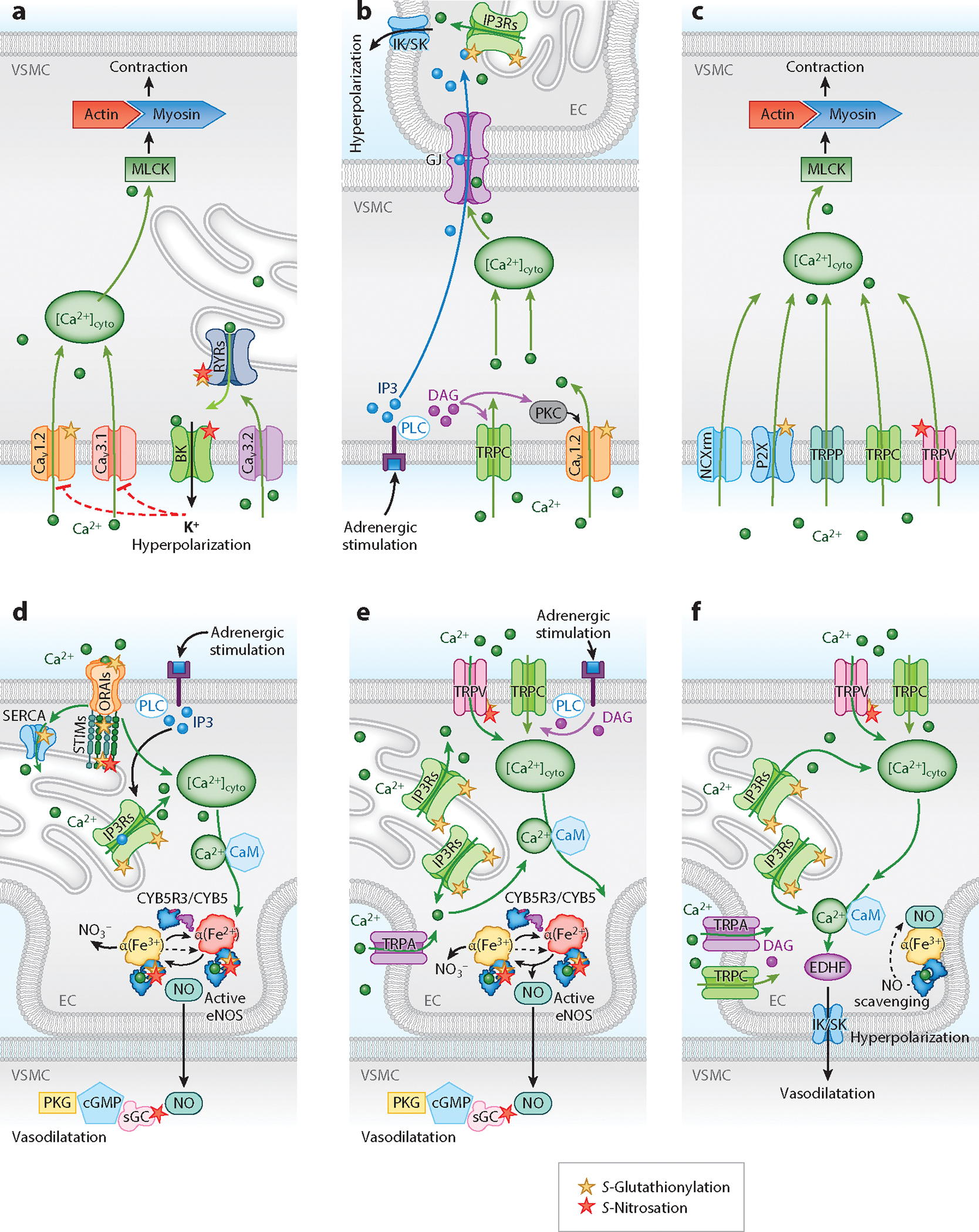
Ca^2+^ entry through voltage-gated Ca^2+^ channels (Ca_V_1.2 and Ca_V_3.1) triggers the VSMC contractile machine through the activation of MLCK to promote VSMC contraction. (*a*) Activation of Ca_V_3.2 channels stimulates RyRs through CICR to trigger Ca^2+^ release from the SR/ER, potentiating BK channel–mediated hyperpolarization and vasodilatation. The red dotted line indicates inhibition of Ca_V_1.2 and Ca_V_3.1 channel function by hyperpolarization. (*b*) Adrenergic stimulation induces IP3 and DAG formation by PLC. DAG activates TRPC channels and PKC, which phosphorylates the Ca_V_1.2 channel to increase [Ca^2+^]_cyto_ and induce vasoconstriction in VSMCs. IP3 and Ca^2+^ can diffuse from VSMCs to the EC through the GJs of the myoendothelial junctions to increase EC [Ca^2+^]_cyto_ and limit vasoconstriction by activating IK/SK channels. (*c*) Activation of NCXrm and P2X and TRPP, TRPC, and TRPV channels increases VSMC [Ca^2+^]_cyto_ to induce vasoconstriction through the activation of MLCK/actin-myosin. (*d*) IP3R-mediated SR/ER Ca^2+^ release is induced by CICR or adrenergic stimulation through the generation of IP3 by PLC. The subsequent decrease in [Ca^2+^]_SR/ER_ induces SOCE (mediated by STIM and ORAI) and ER store refilling (facilitated by the activity of SERCA) to increase [Ca^2+^]_cyto_ for Ca^2+^–CaM-dependent activation of eNOS and generation of NO. NO diffuses to the VSMCs and causes vasodilatation through the activation of the NO-sGC-cGMP-PKG pathway. (*e*) Activation of TRPV, TRPC, and TRPA channels through mechanical stimuli and/or adrenergic activation of PLC and generation of DAG and subsequent activation of SR/ER Ca^2+^ release (IP3R) increase [Ca^2+^]_cyto_ to activate eNOS and cause VSMC relaxation through the production of NO. (*f*) Ca^2+^ influx via TRPC, TRPV, and TRPA channels and/or Ca^2+^ release from the SR/ER through IP3Rs activates IK/SK channels via Ca^2+^–CaM. The activation of the IK/SK channels (EDHF) causes EC hyperpolarization and VSMC relaxation. eNOS-derived NO is scavenged by α-globin in the microcirculation. Abbreviations: Ca^2+^–CaM, calcium-calmodulin; [Ca^2+^]_cyto_, cytosolic Ca^2+^ concentration; [Ca^2+^]_SR/ER_, sarcoplasmic/endoplasmic reticulum Ca^2+^ concentration; Ca_V_1.2, voltage-gated Ca^2+^ channel 1.2; Ca_V_3.1, voltage-gated Ca^2+^ channel 3.1; cGMP, cyclic guanosine 3′,5′-cyclic monophosphate; CICR, Ca^2+^-induced Ca^2+^ release; CYB5, cytochrome B5; CYB5R3, cytochrome B5; reductase 3; DAG, diacylglycerol; EC, endothelial cell; EDHF, endothelial-derived hyperpolarizing factor; eNOS, endothelial nitric oxide synthase; GJ, gap junction; IK/SK, intermediate and small conductance Ca^2+^-activated K^+^ channels; IP3, inositol 1,4,5-trisphosphate; IP3R, IP3 receptor; MLCK, myosin light chain kinase; NCXrm, Na^+^/Ca^2+^ exchangers in reverse mode; NO, nitric oxide; ORAI, calcium release–activated calcium channel protein; P2X, purinergic P2X; PKG, protein kinase G; PLC, phospholipase C; RyR, ryanodine receptor; SERCA, SR/ER Ca^2+^ ATPase; sGC, soluble guanylyl cyclase; SOCE, store-operated Ca^2+^ entry; STIM, stromal interaction molecule; TRP, transient receptor potential channel; TRPA, ankyrin-rich protein TRP subfamily; TRPC, canonical TRP subfamily; TRPP, polycystic TRP subfamily; TRPV, vanilloid TRP subfamily; VMSC, vascular smooth muscle cell.

One of the major distinctions between the two VGCCs is that, unlike LTCCs, TTCCs are not associated with auxiliary subunits (32). TTCC currents are mediated by ion-conducting α_1_G,α_1_H, and α_1_I subunits encoded by Ca_V_3 (Ca_V_3.1–3) genes, the expression of which has been detected in all arteries (33). Regulation of TTCCs occurs via protein kinases, low concentrations of metal ions (Ni^2+^, Zn^2+^), and ROS and RNS (34, 35). NO-dependent cGMP/PKG signaling inhibits TTCC currents in cerebral arteries (35). H_2_O_2_ was shown to inhibit Ca_V_3.2 currents, likely through the oxidization of histidine 191 in the channel’s extracellular IS3–IS4 domains (36). Amelioration of Ca_V_3.2 currents and Ca_V_3.2-ryanodine receptor (RyR)-BK signaling, which promote vasoconstriction, occurs following angiotensin 2 activation of NADPH oxidase (36). TTCC activation and myogenic constriction have also been shown to be inhibited by NO-triggered cGMP/PKG signaling and PKA activation in rat cerebral arteries (35, 37). There is likely cross talk between the ROS- and RNS-mediated regulation of both LTCCs and TTCCs to modify vascular tone (Figure 1a).

Signaling for VSMC contraction is also regulated by nonvoltage-gated Ca^2+^ channels through purinergic P2X receptor (P2XR) activation, transient receptor potential (TRP) channels, store-operated Ca^2+^ entry (SOCE) channels, Na+/Ca^2+^ exchangers in reverse mode (NCXrm), and Ca^2+^ release from intracellular stores (Figure 1c). Because an extensive review on the VSMC and EC Ca^2+^ signaling was recently published by Ottolini & Sonkusare (38), this review only covers the channels that are known or suspected to be redox regulated.

Purinergic signaling controls vascular remodeling and resistance. P2XRs are ubiquitously expressed nonselective cation channels that transfer monovalent and divalent cations released from perivascular nerve terminals at neuromuscular junctions or by pannexins in the VSMC/EC membranes (39, 40). Purinergic signaling is subject to modulation, as shown by studies wherein ATP-induced P2XR signaling increased [Ca^2+^]_cyto_ in preglomerular VSMCs (41). Subsequent studies showed that H_2_O_2_ potentiated P2XR through the oxidation of its Cys430 thiol group in HEK293 cells (39).

In addition to purinergic channels, TRP channels are nonvoltage-gated cation channels regulating contractility and proliferation in VSMCs (42). Their major role is to promote global or local [Ca^2+^]_cyto_ transients that induce membrane depolarization through ion channel activation. The TRP channel family consists of six subfamilies: canonical (TRPC), melastatin (TRPM), mucolipins (TRPML), vanilloid (TRPV), polycystic (TRPP), and ankyrin-rich protein (TRPA) (43). The most abundant TRP channels in VSMCs are TRPV (1 and 4), TRPC (1, 3 and 6), and TRPP (1 and 2).

Most vascular beds have TRPV channels in both VSMCs and ECs. TRPV channels generally conduct more Ca^2+^ than Na^+^ and are regulated by temperature, mechanical stimuli, or neurohormonal signals. The main endogenous regulators of TRPV1 channel activity are PKC, [Ca^2+^]_cyto_, and calcineurin (44). TRPV4 channels in VSMCs can promote both vasodilatation and vasoconstriction depending on the signaling pathways and the vascular bed (38). In VSMCs of mesenteric and cerebral arteries, TRPV4 mediated the Ca^2+^ influx-induced activity of RyR-BK signaling and hyperpolarization, promoting vasodilatation. TRPV4 channels have been shown to counteract angiotensin 2–induced vasoconstriction in cerebral arteries through enhancement of AKAP150 and PKCα interactions that phosphorylate TRPV4 channels in a proximity-dependent manner (45). By contrast, upregulation of TRPV4 channels in murine pulmonary arteries has been shown to enhance contractility and a hypertensive phenotype (46). These findings indicate that TRPV4 channels regulate systemic and pulmonary vasculature differently. While ROS-related activation of TRPV4 channels has been observed with H_2_O_2_-induced phosphorylation of Ser824 and inhibition by RNS through NO-induced *S*-nitrosation of Cys853, redox regulation of TRPV channels is more established in ECs and may be cell type dependent (47, 48).

TRPM channels are Ca^2+^ activated, Ca^2+^ impermeable, and nonselective. As cation channels, they are key regulators of pressure-induced depolarization of VSMC membranes (49). TRPMs are expressed in VSMCs and mostly transfer monovalent ions. Increased [Ca^2+^]_cyto_ interacts with TRPM4 by calmodulin (CaM) binding, which facilitates channel membrane depolarization and LTCC activation in VSMCs (49). Redox modification of Cys1093 of TRPM4 channels caused reduced desensitization, possibly through increased CaM binding. H_2_O_2_ stimulated TRPM4 channels in human umbilical vein cells and through an as-yet-unknown mechanism increased its expression levels (50).

TRPC channels are nonselective cation channels with three types (TRPC1, 3, and 6) expressed in VSMCs. These channels are permeable to monovalent and divalent cations in an isoform-specific manner. TRPC3 and TRPC6 are both activated by diacylglycerol (DAG) in a PKC-independent manner (51). It has been found in cerebral arteries that the C-terminal tail of TRPC3 interacts with the N-terminal of inositol 1,4,5 trisphosphate receptor (IP3R) 1, which causes TRPC3 channel activation and membrane depolarization (52). Depolarization of VSMCs activates VGCCs, leading to vasoconstriction. For TRPC6, activity is increased by α-adrenergic stimulation and DAG-PLC signaling (Figure 1b). In the context of mechanical stimulation, TRPC6 channels can also participate in myogenic vasoconstriction. On a different note, there is controversy surrounding the role of TRPC1 and other SOCE proteins, such as stromal interaction molecule 1-calcium release–activated calcium channel protein 1 (STIM1–ORAI1), in VSMC Ca^2+^ signaling. TRPC1 has been reported to participate in SOCE in VSMCs, which is central to cellular Ca^2+^ homeostasis, and STIM–ORAI has been shown to mediate SOCE in rat aorta and pulmonary arteries. Knockdown of either STIM1 or ORAI1 has been shown to reduce SOCE and Ca^2+^ release–activated Ca^2+^ channel currents in response to Ca^2+^ store depletion or hypoxia, impairing VSMC migration and proliferation (53). Silencing of TRPC channels (TRPC1, 4, or 6) had no effect on SOCE in VSMCs (53). Although normal SOCE has been reported in aorta and cerebral arteries of TRPC1 null mice, anticipated induction of SOCE by thapsigargin was inhibited by STIM1 silencing (54). Additionally, native VSMCs expressed low levels of STIM–ORAI (55). In contrast, a molecular complex between TRPC1 and STIM1 was identified as the main regulator of SOCE in pulmonary arterial smooth muscle cells (56). These data suggest that the exact role of SOCE and its regulation by nonvoltage-gated channels need further study in the cardiovascular system. Redox regulation of STIM–ORAI IP3Rs by H_2_O_2_ are discussed in the next section.

Cytosolic Ca^2+^ transients are not only generated by transport of extracellular Ca^2+^ across the plasma membrane but also can be increased by second messenger-induced Ca^2+^ mobilization from intracellular stores by redox sensitive high-conductance cation channels (IP3Rs and RyRs). RyR is activated by Ca^2+^-induced Ca^2+^ release (CICR) or the binding of cyclic ADP ribose, and its activity is increased by thiol modification, such as *S*-glutathionylation and *S*-nitrosation (57), which can occur at the RyR Cys3635. Substitution of Cys3635 with alanine was found to cause loss of sensitivity to L-type Ca^2+^ channel voltage-gated activation, highlighting the redox sensor nature of Cys3635 in its role as mediator of RyR-dependent Ca^2+^ release.

Furthermore, a recent study proposed a close interplay between TRP channels and IP3Rs in cerebral arteries, wherein increased intraluminal pressure-induced TRPC6-mediated Ca^2+^ influx enhanced IP3R activity (58). SR/ER Ca^2+^ mobilization by IP3Rs was instrumental in activating TRPM4 channels for membrane depolarization and vasoconstriction. Ca^2+^ release from the SR/ER is inducible by activation of TRPML1, RyRs, or IP3Rs. Notably, α_1_-adrenergic signaling has been found to increase intracellular IP3, triggering [Ca^2+^]_cyto_ signaling and VSMC constriction.

### Phase 2: Redox Regulation of Calcium Signaling at the Myoendothelial Junction

Following receptor-dependent or -independent IP3 activation,Ca^2+^ release and vasoconstriction, resistance arteries and arterioles are known to subsequently release vasodilators to modulate the contractile response. A seminal study by Dora et al. (59) uncovered this phenomenon following phenylephrine or potassium chloride stimulation of arterioles, which triggered a rise in VSMC and EC calcium, leading to contraction and increased NO in arterioles. This feedback mechanism was proposed as a mechanism for arterioles to buffer the contractile response through a process thought to involve VSMC-to-EC signal transmission through the MEJ. Serving as a connective device between ECs and VSMCs, the MEJ was considered at the time to be a potential microsignaling domain due to its enrichment of ER, caveolae, and mitochondria (3). High-resolution electron microscopy revealed that EC and VSMC membranes are juxtaposed at the MEJ, opening up the possibility that gap junctions (GJs) may be positioned at these contact sites (60). Using a transwell coculture model system of ECs and VSMCs, Isakson & Duling (61) established that EC and VSMC contact at the MEJ stimulates organized GJ formation. Composed of connexins, which form a hexameric channel allowing small molecules less than 1 kDa to pass between cell types, these MEJs allowed trafficking of IP3 from VSMCs to ECs, causing activation of IP3R1 and release of local calcium at the MEJ (62). The MEJs are enriched with eNOS, an enzyme regulated by Ca^2+^, indicating that compartmentalized stimulation of IP3-mediated Ca^2+^ signaling activates eNOS to provide a feedback mechanism to control vasoconstriction (63). The discovery of eNOS-derived NO signaling at the MEJ opened up the potential for a unique microdomain composed of redox switches.

**Figure 2:**
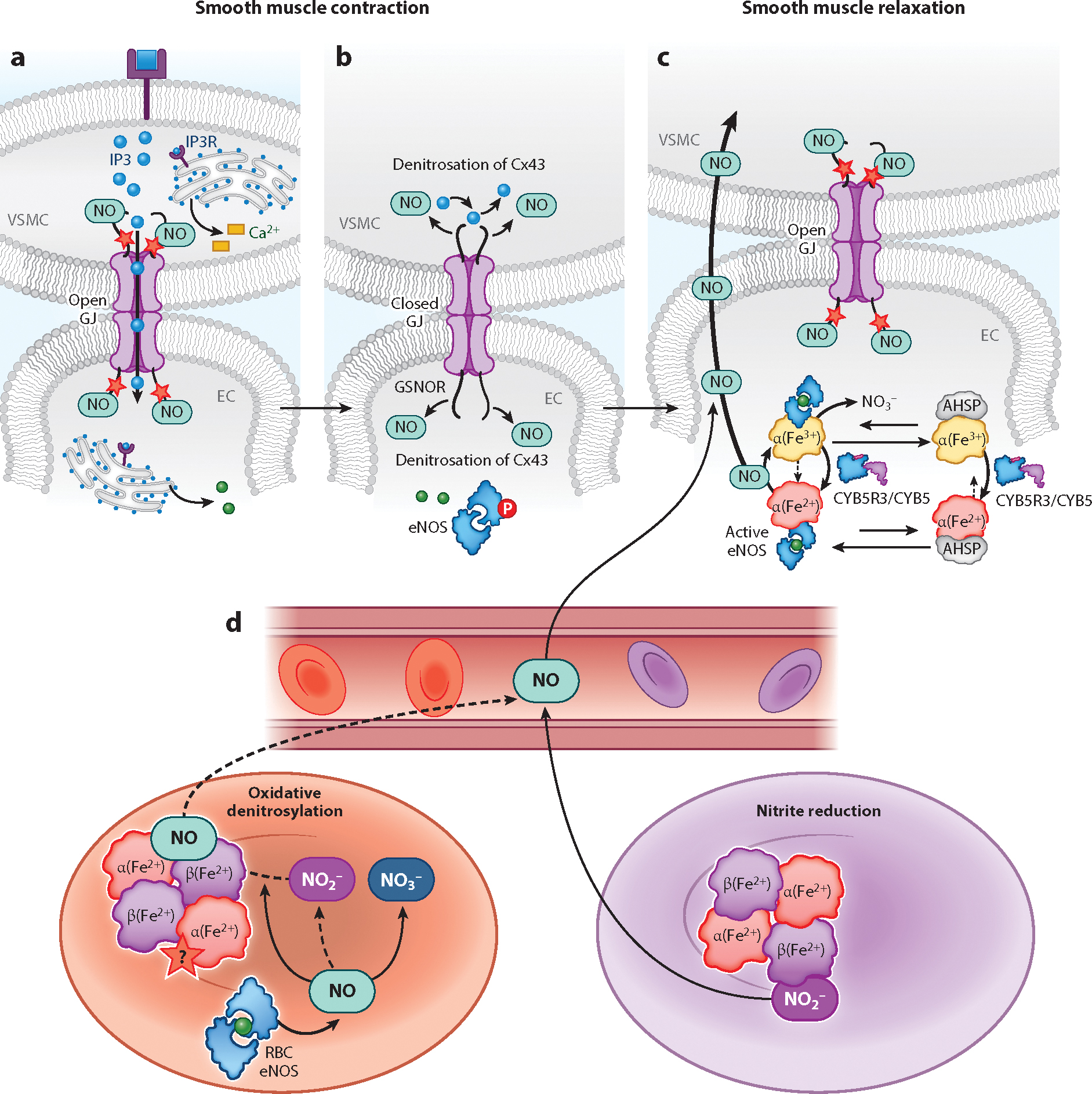
(*a*) *S*-Nitrosation of Cx43 facilitates the open state of GJs at the myoendothelial junctions to allow the intercellular exchange of signaling molecules. (*b*) IP3-induced Ca^2+^ release from the ER activates eNOS through phosphorylation (P *circled in red*). Denitrosation of Cx43 by GSNOR closes GJs and limits the diffusion of IP3. (*c*) NO generated by the activity of eNOS, which is mediated by the changes in the α-globin heme redox state. The NO generated during eNOS activity can diffuse from the EC to VSMC to cause vasodilatation and is important in maintaining the open probability of GJs through the *S*-nitrosation of Cx43. α-Globin is stabilized by AHSP before and complexes with eNOS to facilitate NO production. The redox state of the α-globin heme is regulated by CYB5R3 or eNOS. *S*-Nitrosation is marked with a red star. (*d*) In oxygenated RBCs (*red*), eNOS generates NO that can ultimately participate in vasorelaxation to regulate blood flow and blood pressure. Redox reactions of NO with oxygen, oxyhemoglobin, and deoxyhemoglobin result in nitrite NO_2_^−^, NO_3_^−^, and Fe^2+^-nitrosyl-hemoglobin. During gas exchange, the RBC partially deoxygenates (*purple*), and diffusible molecules that can signal for vessel relaxation are generated. NO_2_^−^ undergoes a reductive reaction with deoxyhemoglobin, releasing diffusible NO as the oxygen tension approaches the point where 50% of the RBC hemoglobin has become deoxygenated. Reactive intermediates (NO_2_^−^ and H_2_O_2_) formed in the process also facilitate the release of NO from Fe^2+^-nitrosyl-hemoglobin (oxidative denitrosylation) and participate in radical–radical interactions that generate the diffusible N_2_O_3_ for possible RBC export. The red star marks suspected *S*-nitrosation (*S*-nitrosothiol modification of hemoglobin), which is distinct from Fe^2+^-nitrosyl-hemoglobin formation. Abbreviations: AHSP, α-globin stabilizing protein; Cx43, gap junction–forming vascular connexin; CYB5, cytochrome B5; CYB5R3, cytochrome B5 reductase 3; EC, endothelial cell; eNOS, endothelial nitric oxide synthase; GJ, gap junction; GSNOR, *S*-nitrosoglutathione reductase; H_2_O_2_, hydrogen peroxide; IP3, inositol 1,4,5-trisphosphate; IP3R, IP3 receptor; N_2_O_3_, dinitrogen trioxide; NO, nitric oxide; NO_2_^−^, nitrite; NO_3_^−^, nitrate; RBC, red blood cell; VMSC, vascular smooth muscle cell.

Ca^2+^ signaling and redox events in VSMCs are followed by diffusion of Ca^2+^ and IP3 to ECs through MEJs. Three out of the four known GJ-forming vascular connexins (Cx37, Cx40, and Cx43) are found at MEJs to facilitate second messenger and electrical signaling (63, 64). Connexins at the MEJ are regulated by posttranslational modification, such as phosphorylation of specific serine (Ser) residues or through *S*-nitrosation/denitrosation. Modification of Cx43 at Cys271 controls GJ permeability through an eNOS/NO and *S*-nitrosoglutathione reductase (GSNOR)-dependent mechanism, highlighting an important redox switch controlling cross-communication between ECs and VSMCs (63) (Figure 2a,b).

Transfer of IP3 through the MEJs induces IP3-mediated increases in endothelial [Ca^2+^]_cyto_ through IP3Rs and TRPV4 channels, activating vasodilatory signaling by NO and endothelial-derived hyperpolarizing factor (EDHF). Interestingly, NO plays only a marginal role in agonist-induced vasodilatation compared to EDHF in resistance arteries (65). Vascular EDHF is thought to be a substance or electrical signal generated and released by healthy ECs for effecting VSMC hyperpolarization and relaxation. Although the exact identity of EDHF (e.g., K^+^, sulfur, eiconsanoids, C-type natriuretic peptide) and its mechanism of action still engender debate, the literature suggests that K^+^ is a common factor in EDHF signaling in the vasculature. EDHF signaling for vasodilatation increases as vessel diameters decrease—namely at sites of blood flow and blood pressure regulation—and relies on activation of BK channels. The switch from NO-to EDHF-based signaling cannot be explained by the lack of eNOS expression in the microcirculatory vessels (66). The initiation of EDHF signaling in ECs for VSMC hyperpolarization and relaxation relies on the activation of BK channels. The mechanism for differences in regulation of vasodilation in resistance arteries versus conduit arteries may be due to NO scavenging (67) (Figure 1e–f).

Recently α-globin was found to be expressed and concentrated at the MEJs in arteriolar ECs (68). In contrast to RBCs, where α-globin exists in a stable complex with β-globin, a recent study showed that EC α-globin undergoes stabilization when chaperoned by α-globin stabilizing protein (AHSP) prior to complexing with eNOS for regulation of NO diffusion from ECs to VSMCs (69) (Figure 2c). Disruption of the α-globin-eNOS complex, either by a peptide mimetic or genetic knockdown of either the α-globin or *AHSP* gene, has been shown to increase NO signaling and VSMC relaxation.

The redox state of the α-globin heme iron determines whether it scavenges NO or permits its diffusion. Reduced heme iron (Fe^2+^) promptly reacts with NO to form iron-nitrosyl-hemoglobin (Fe^3+^-NO), limiting its diffusion to VSMCs. However, oxidized heme iron (Fe^3+^) reacts very slowly with NO, allowing its intercellular diffusion to induce VSMC relaxation and regulation of vascular tone. The redox state of α-globin is controlled by cytochrome B5 reductase 3 (CYB5R3), which is a flavoprotein that complexes with eNOS and α-globin (70). In addition to CYB5R3, eNOS has also been shown to regulate α-globin reduction (69). Thus, the redox control of α-globin, with the reduced heme scavenging NO and the oxidized heme allowing NO diffusion, serves as a redox controlled gate for NO diffusion.

Other globins are expressed in nonerythroid cells and have been suggested to control NO and H_2_O_2_ bioavailability, thus controlling vascular tone. Neuroglobin (Ngb) and cytoglobin (Cygb) are different from hemoglobin and myoglobin, as they exhibit hexacoordinate heme binding, with both a proximal and distal heme pocket histidine binding to the heme iron. Interestingly, they possess two surface thiols that, when oxidized to form an intramolecular disulfide, allosterically promote a weakening of the distal histidine bond and increase ligand affinity and reactivity of the heme (71–73). For both Ngb and Cygb these cysteines thus serve as redox sensors that, when oxidized, increase ligand affinity and the reaction rate with molecules such as nitrite, which is reduced to form NO. In the case of Cygb there are subpopulations ranging from fully reduced thiols, single thiol oxidation, and intramolecular disulfide formation that determine heme-binding properties by modulating the histidine-heme affinity and ligand binding (71). The redox modulation of thiol oxidation state and heme-ligand binding is sensitive to physiological levels of H_2_O_2_, with a functional midpoint redox potential for the native Cygb intramolecular disulfide bond of −189 ± 4 mV, a value within the normal range of intracellular redox potentials (71). In the case of Ngb, mutation of either Cys55 or Cys46 to alanine stabilizes the six-coordinate structure and slows the reaction with nitrite (72). These results support the hypothesis that Cys38 and Cys83 on Cygb serve as sensitive redox sensors that modulate the Cygb distal heme pocket reactivity and ligand binding.

While the physiological function of both Ngb and Cygb remains uncertain, they both have lower expression levels than myoglobin and hemoglobin, suggesting they play a limited role in O_2_ regulation (74). A number of functional roles have been proposed, including (*a*) NO scavenging by the deoxygenation reaction $\left({\text{Fe}}^{2+}-{\text{O}}_{2}+\text{NO}\to {\text{Fe}}^{3+}+{\text{NO}}_{3}^{-}\right)$ to increase vascular tone (75), (*b*) nitrite reduction under deoxygenated conditions to promote NO signaling $\left({\text{Fe}}^{2+}+{\text{NO}}_{2}^{-}+\right.\left.{\text{H}}^{+}\to {\text{Fe}}^{3+}+\text{NO}+{\text{OH}}^{-}\right)$ (76), superoxide scavenging $\left(\text{CygbF}{\text{e}}^{3+}+2{\text{O}}_{2}\cdot \overset{-2H+}{\to }{\text{CygbFe}}^{3+}+{\text{H}}_{2}{\text{O}}_{2}+{\text{O}}_{2}\right)$ (77), and (*d*) peroxidase activity that can catalyze the peroxidation of anionic phospholipids (including phosphatidylinositol phosphates), yielding mono-oxygenated molecular species (78). The surface thiol redox sensor–regulated control of these reactions most likely modulates vascular tone, responses to inflammatory and oxidative injury, and remodeling and cellular apoptosis (75, 79). Similar to hemoglobin intracellular α-globin and sGC, additional enzymatic redox control is determined by the oxidation and reduction of the heme by the cytochrome B5 reductase system (80, 81).

### Phase 3: Control of Nitric Oxide in Smooth Muscle and Activation of Soluble Guanylyl Cyclase

The primary NO receptor in VSMCs is sGC. To reach optimal catalytic activity, sGC requires a heterodimeric structure formed from α and β subunits. The sGC protein has two types of α (α1 and α2) and two types of β (β1 and β2) subunits (82).The β2 subunit is expressed at the lowest level of all the subunits, resulting in the most common heterodimers being α1β1, followed by α2β1. It has been shown that VSMC-specific loss of sGCβ1 causes hypertension, and global deletion of sGCβ1 causes gut dysmotility and death (83).

The sGCβ subunit contains a heme region that can interact with NO, causing the heme-iron atom to be cleaved from the H105 residue of sGCβ (84). The binding of NO also facilitates conformational changes in the coiled-coil domains of both α and β subunits, resulting in the alignment of their catalytic sides to form a 5′-GTP binding motif. Heat shock protein 90 (HSP90) has been shown to drive heme into the heme-deficient sGCβ1 form during maturation, the dissociation of which is promoted by NO binding to support heterodimer formation with sGCα1 for an enzymatically active protein (85).

The redox state of the heme iron in sGC is central to NO signaling for cGMP production by the enzyme. When the sGC heme iron is reduced (Fe^2+^), it binds NO, and cGMP production occurs. When the sGC heme iron is oxidized (Fe^3+^), it only weakly binds NO, and cGMP production is blunted (86). Prior work has shown that CYB5R3 regulates the sGC heme redox state, particularly under oxidative stress conditions (87, 88). Oxidized sGC heme iron needs to be reduced back to its Fe^2+^ state to sense NO (70), which our work has shown is the role of CYB5R3 in VSMCs. It has yet to be determined whether CYB5R3 serves as a direct modulator of cysteine redox states during oxidative stress or affects *S*-nitrosation.

A key redox switch in regulating sGC function is disulfide bond formation between oxidation sensitive thiols, such as Cys489 and Cys571 of sGCβ1 (89). Treatment with thiol blockers (CdCl_2_, hydroxymercuribenzoate, *N*-ethylmaleimide, etc.) has been shown to decrease sGC responsiveness to NO (90). *S*-Nitrosation of specific cysteines in the β1 subunit has been shown to negatively regulate sGC activity in the presence of NO (91). In the presence of thioredoxin 1 (Trx1), *S*-nitrosated cysteines are reduced, allowing protein disulfide isomerase to form disulfide bonds between Trx1 Cys73 and sGCα1 Cys609. This interplay between Trx1 and sGC promotes the production of cGMP under high NO conditions, as shown by significantly decreased cGMP production with nitrosocysteine treatment during Trx1 inhibition and rescue of cGMP production during Trx1 overexpression (92).

Following the NO activation of sGC, protein kinase G1 (cGMP-activated protein kinase) is activated, eliciting relaxation of VSMCs through the inactivation of the myosin contractile machinery (93). There are two known splice variants of PKG1 (α and β) with different cGMP-binding affinity and phosphorylation substrate selectivity (94). PKG1α has redox-sensitive motifs that factor into the kinase’s function under pathological conditions. Disulfide bridge formation at the redox-sensitive Cys42 confers functionality on the kinase. Residues Cys117/Cys195 and Cys312/Cys518 within the high- and low-affinity cGMP-binding sites have been suggested to be sensitive to oxidative activation when enlisted for disulfide bridge formation (95). Studies with Cys42Ser mutant mice have revealed that interference with disulfide bond formation can yield diverging downstream effects for PKG1α. At baseline, Cys42Ser mutant mice display hypertension, less acetylcholine-dependent dilatation, decreased PKG activity after pro-oxidant exposure (H_2_O_2_), and impaired myocardial relaxation and contractility (96). However, when subjected to aortic constriction, Cys42Ser mutant mice were shown to be more resistant to severe pathology and death (97). These findings highlight the possible roles of cGMP signaling in certain pathological conditions.

## ENDOTHELIAL TO SMOOTH MUSCLE CELL CROSS TALK AND CONTROL OF VASODILATION

### Calcium Regulation of Nitric Oxide–Mediated Vasodilation

EC-mediated vasodilatation undergoes differential regulation determined by the size of the artery or arteriole. Whereas vasodilation in conduit arteries (i.e., aorta) occurs through eNOS-derived NO, vasodilation in systemic resistance arteries and arterioles can be driven by several distinct pathways: NO, EDHF, prostaglandins, H_2_O_2_, and hydrogen disulfide (H_2_S_2_). In contrast to VSMC [Ca^2+^]_cyto_, EC [Ca^2+^]_cyto_ facilitates vasodilatation through eNOS-derived NO. Signaling molecules cross over from VSMCs to ECs, activating cellular Ca^2+^ signaling and NO generation by eNOS. NO synthesis by eNOS requires its precursor l-arginine, the cofactor tetrahydrobiopterin (BH4), flavin dinucleotide, flavin mononucleotide, CaM, and heme iron. For eNOS to produce NO, its oxygenase and reductase domains must be heterocomplexed into a dimer mediated by HSP90 (98). The eNOS heterodimer can become uncoupled in the absence of sufficient l-arginine or BH4, leading the oxygenase domain to preferentially produce superoxide (O_2_^−^) and contribute to oxidative stress in certain pathologies (98). Uncoupling of the eNOS enzyme also occurs with *S*-glutathionylation of Cys689 and Cys908 (99).

eNOS is tightly regulated, by both its location in caveolae and posttranslational modifications. The localization of eNOS in caveolae allows its interaction with the scaffold protein caveolin-1, which keeps it inactive. Posttranslational modifications also regulate eNOS activity in a manner that depends on the site and type of modification. eNOS is activated through phosphorylation of Ser1177 by PKA, PKB, AMPK, CaMK, and ERK1 and 2, and inhibited by the phosphorylation of tyrosine (Tyr) 495 by Rho kinase and PKC (5, 99, 100). In addition, phosphorylation of Tyr657 by tyrosine kinase lowers eNOS activity, and phosphorylation of Tyr81 by Src facilitates NO production (99). Redox switches are important for regulating eNOS activity; *S*-nitrosation of Cys94 and Cys99 or glycosylation of Ser1177 blunts enzyme activity, while acetylation of Lys610 and deacetylation of Lys497 and Lys507 increase enzyme activity (99).

In response to extracellular signals, EC cytosolic Ca^2+^ signaling is mostly initiated by Ca^2+^ release from intracellular stores through large,high-conductance Ca^2+^ channel IP3Rs and sustained by SOCE or TRP channels to induce eNOS activation or EDHF. The IP3Rs that participate in Ca^2+^ release come in three isoforms (IP3R1–3), all of which are present in ECs. IP3Rs facilitate Ca^2+^ mobilization from the SR/ER upon stimulation by GPCRs or tyrosine kinase receptors on the plasma membrane or by Ca^2+^ through CICR (101, 102). Endothelial GPCRs recruit phospholipase C (PLC) β2 and β3 to transform phosphatidylinositol 4,5-bisphosphate into DAG and IP3, while PLCγ1 couples with tyrosine kinase receptors to promote IP3 generation (103) (Figure 1d). Sensitization of IP3R channel activity is tightly regulated by oxidation of specific thiols. Mammalian IP3R1 has 60 thiol groups, a significant fraction of which are sensitive to oxidative posttranslational modifications (104). IP3R1 has been shown to have cytosolic (Cys292 and Cys1415) and ER lumen cysteines (Cys2496 and Cys2533) that are oxidized under basal conditions, and a handful of N-terminal cysteines (Cys206, Cys214, and Cys1397) that can be oxidized by H_2_O_2_ (Cys206, Cys767, and Cys1459) and thimerosal (104). *S*-Glutathionylation has been shown to increase IP3R activity in ECs after treatment with a thiol oxidizing agent (diamin) (105). Apart from IP3R1, the other two IP3R isoforms have undergone limited characterization in terms of redox regulation (104). IP3R1 and IP3R2 were shown to be more sensitive to redox modification to alter ligand sensitivity, while IP3R3 is relatively insensitive (106). H_2_O_2_ can also drive IP3-induced SR/ER Ca^2+^ release by directly acting on PLCγ1 and/or by stimulating IP3Rs. Indeed, H_2_O_2_ has been shown to promote IP3 production in aortic and mesenteric artery ECs (107). There is also evidence for exogenous H_2_O_2_ induction of a dose-dependent oscillatory [Ca^2+^]_cyto_ increase in human aortic ECs (108). These oscillations were shown to be completely dependent on Ca^2+^ release from the ER, with no dependence on Ca^2+^ flux through the plasma membrane. By contrast, human umbilical vein ECs respond differently to H_2_O_2,_ undergoing a marked reduction of ER Ca^2+^ concentration ([Ca^2+^]_ER_) (109). Of note, it has been speculated that H_2_O_2_ depolarization of mitochondria may inhibit IP3R-induced Ca^2+^ release in vascular ECs.

The SR/ER is the most important and largest intracellular Ca^2+^ store. Here, the SR/ER Ca^2+^ ATPase (SERCA) mainly regulates intracellular Ca^2+^ concentration and refills ER stores during SOCE (Figure 1d). SERCA is prone to increased activation by *S*-glutathionylation by peroxynitrite (ONOO^−^) generated from NO under conditions of oxidative stress (110). In atherosclerosis, the Cys674 site of SERCA was found to undergo irreversible oxidation to sulfonic acid, preventing NO-induced VSMC relaxation (111). Although modification of Cys674 by sulfonic acid has been shown in large vessels, it is unclear whether the same modification happens in the microcirculation.

After Ca^2+^ release, ER stores need to be refilled, and multiple mechanisms in the plasma membrane exist. STIM1 and STIM2, which sense ER Ca^2+^ levels, work together with ORAI (ORAI 1–3) Ca^2+^ channels to drive SOCE (Figure 1d). SOCE is primarily facilitated by changes in the ER Ca^2+^ content; however, it is also regulated through posttranslational modifications of STIM and ORAI proteins. Two STIM1 cysteines (Cys49 and Cys56) have been identified as ROS/RNS sensors that are modifiable by *S*-glutathionylation and *S*-nitrosation, affecting the stability of the EF-hand domains of calcium-binding proteins (112–114). *S*-Glutathionylation of Cys49 and Cys56 destabilizes the EF-hand domain, decreasing affinity for Ca^2+^ and causing STIM1 and ORAI1 activation (115). *S*-Nitrosation of Cys49 and Cys56 stabilizes the EF-hand domain, causing SOCE inhibition (113, 114). Little is known about the other three STIM1 cysteines; however, it has been suggested that the cytosolic Cys437 residue may serve as an oxidative stress sensor that drives conformational changes in ORAI1 before SOCE (112). For the STIM2 protein there are 15 cysteines, with four luminal cysteines corresponding to the ones found in STIM1, and 11 cytosolic cysteines that are unique (112). Cys313 was identified recently as a ROS sensor in STIM2. Oxidation of Cys313 interferes with STIM2 activation and inhibits SOCE, most likely through altering STIM2–STIM2 interactions (116). Redox modification of ORAI proteins can also occur (112). Oxidation of ORAI1 and ORAI2 at Cys195 has been shown to inhibit SOCE in various models. While ORAI3 does not contain a Cys195 site, it has other cysteine sites that may allow it to be oxidatively regulated. H_2_O_2_ treatment has been shown to prevent ORAI1–ORAI1 interactions, suggesting that Cys195 oxidization can prevent ORAI1 subunit interactions, causing a deficiency of pore-forming units. By contrast, H_2_O_2_ treatment increases STIM1–ORAI1 interactions (112). While the different redox modifications and their effect on SOCE have been extensively studied in the past, we lack an understanding of the role of different cellular oxidants and reductants in SOCE regulation.

Generation of [Ca^2+^]_cyto_ transients in ECs can also occur via NCXrm. NCXrm is known to participate in vasodilatation through acetylcholine-stimulated activation of EC muscarinic receptor signaling (117). An association between NCX and eNOS has been shown to happen in caveolae. NCX-mediated increases in [Ca^2+^]_cyto_ are coupled to both NO production and intermediate and small conductance Ca^2+^-activated K^+^ (IK/SK) channel activation in ECs. ROS-stimulated activation of NCX is suspected to occur via phosphorylation of the intracellular loop of NCX by Ser/Thr kinases (PKA or PKC) (118).

### Endothelium-Derived Hyperpolarization

In the microcirculation EDHF is the main driver of vasodilatation. IK/SK channels play a crucial role in EC control of VSMC relaxation (Figure 1f). Endothelial IK/SK channels become activated upon interacting with Ca^2+^–CaM, resulting in hyperpolarization of the EC membrane and VSMC relaxation. Influx of extracellular Ca^2+^ for these signaling events is mediated through activation of various TRP channels.

TRPA1 is a crucial Ca^2+^ influx pathway in ECs. TRPA1 colocalizes with IK channels at the MEJs in cerebral arteries. TRPA1-mediated Ca^2+^ influx activates nearby IK channels to effect EC membrane hyperpolarization and subsequent vasoconstriction. TRPA1 channels also promote SR/ER Ca^2+^ release through IP3Rs (119) (Figure 1e, f). Interestingly, NOX2 has been found localized near TRPA1 channels in cerebral arteries. This suggests that NOX2-generated ROS, which are known to cause peroxidation of membrane lipids and 4-hydroxy-2-nonenal formation, can activate TRPA1 channels (120).

TRPV4 channel activity is regulated by numerous proteins and mechanisms, which can be important given the channel’s key role in mediating EC Ca^2+^ influx for blood pressure regulation. While TRPV4 channels can be activated by mechanical stimuli, it is suspected that they are not really mechanosensors and are mainly activated by signaling pathways that include cytochrome P450 activation or epoxygenases (121).

Under pathological conditions, such as pulmonary hypertension, it has been shown that TRPV4 signaling decreases with NOX1/inducible NOS-generated peroxynitrite in endothelial caveolae (122). In small pulmonary arteries, TRPV4-mediated Ca^2+^ sparks occur in response to ATP activation, promoting eNOS activity and NO release (Figure 1e). NO activates sGC-cGMP-PKG signaling, but the outcome is a lowering of Ca^2+^-dependent cooperative opening of TRPV4 channels in ECs. Hydrogen sulfide (H_2_S) is another gasotransmitter that can signal for vasodilatation, sometimes in collaboration with NO. EC-derived H_2_S has been shown to activate TRPV4 channels via sulfhydration, resulting in increased BK channel currents (123).

TRPC3 has been shown to localize close to IK/SK channels at the MEJs in rat mesenteric arteries (124) (Figure 1f). The TRPC3 channel is DAG sensitive and dependent on EC [Ca^2+^]_cyto_ for controlling proliferation, migration, and tube formation linked to chemical (NO) and/or electrical stimulation during vasorelaxation. Activation of TRPC3 by *tert*-butyl hydroperoxide in pulmonary artery ECs has been shown to cause an NO-selective cation current (125). TRP channels may also form heterocomplexes with other TRP channels. TRPC1 has been found heterocomplexed with TRPV4 to activate eNOS in rabbit mesenteric arteries, causing vasodilatation (126) (Figure 1e). Heteromers of TRPC3 and TRPC4 have been detected in ECs, where they may undergo indirect activation by cellular ROS-induced PLC stimulation (127).

Lastly, the activation of P2X, PIEZO1, TRPP1, and TRPV4 channels can be stimulated to increase [Ca^2+^]_cyto_ in a mechanosensitive manner, such as from blood flow–mediated shear stress on vascular endothelium (23). The result is eNOS activation and NO signaling with downstream activation of IK/SK channels, leading to EC-dependent hyperpolarization and VSMC relaxation.

## THE IMPACT OF OXYGEN AND THE CONTRIBUTION OF RED BLOOD CELLS TO THE REGULATION OF VASCULAR TONE AND BLOOD FLOW

The role of RBCs in hypoxic vasodilatation illustrates the importance of O_2_ sensing and redox signaling to the maintenance of vascular homeostasis. At high O_2_ concentrations, RBC hemoglobin exists in a relaxed configuration (R-state), which has a higher O_2_ affinity, facilitating O_2_ binding from in the lung where O_2_ tension is high. Under low O_2_ conditions in the peripheral circulation in metabolically active tissues, RBC hemoglobin deoxygenates and assumes a tense configuration (T-state),which has a lower affinity for O_2_ and thus facilitates dissociation of O_2_ from hemoglobin for supply to less-oxygenated tissues. In addition to O_2_, the RBC dynamically responds to tissue metabolism via allosteric molecular interactions with proton, 2,3-Diphosphoglycerate (2,3-DPG) (modulated by intracellular glycolytic flux) and CO_2_ and in the intracellular domain of band 3. These interactions shift the hemoglobin P_50_, the partial pressure of O_2_ at which hemoglobin is 50% saturated with O_2_, allowing fine-tuning of O_2_ delivery in concert with tissue O_2_ consumption and metabolism. In addition to the canonical roles of hemoglobin in transporting O_2_, CO_2_, and buffering proton, it has been proposed that the hemoglobin and erythrocytes directly release ATP and NO under allosteric control to facilitate hypoxic vasodilation and signaling. While controversial, these models are elegant in presenting a pathway for direct coupling of hemoglobin-based hypoxic sensing to vasodilation.

### Erythrocyte ATP Delivery Hypothesis

Three mechanisms have been proposed for RBC and hemoglobin-mediated hypoxic vasodilation. The first was proposed by Ellsworth, Sprague, and colleagues (128), who presented evidence that erythrocytes release ATP during deoxygenation, which activates purinergic receptors in endothelium to drive retrograde hypoxic vasodilation. Although this pathway has been understudied, a number of studies have continued to evaluate ATP release from RBCs to modulate vasodilation (129). It is our opinion that this pathway remains very promising, especially in light of recent clinical studies showing that the pharmacological modulation of the red cell isoform of pyruvate kinase (PK-R) can increase intracellular ATP levels (130, 131), potentially opening the door to metabolic therapies that enhance hypoxic vasodilation responses. Clearly, more work is required using modern methodologies to fully confirm this hypothesis.

### *S*-Nitroso-Hemoglobin Hypothesis

The second proposed mechanism is the *S*-nitroso-hemoglobin hypothesis (132). This posits that NO binds to the heme of hemoglobin and then transfers in oxygenated blood the Cys93 residue forming a *S*-nitroso-thiol. The NO is then released from the thiol during hemoglobin deoxygenation, likely by transnitrosation to form a still unidentified intermediate X-NO species that has been proposed to facilitate vasodilation (132). A challenge is that the high concentrations of GSH in red cells favor transnitrosation to GSNO, which does not cross the RBC membrane (only l-cysteine can cross via the L-type neutral amino acid transporter) (133). *S*-Nitroso-hemoglobin can be synthesized by incubation of high concentrations of NO with RBCs and by treatment of RBCs with *S*-nitroso-cysteine. Once formed, these RBCs can promote vasodilation, but this does not appear to be O_2_-dependent (134). Furthermore, the chemical mechanism of NO binding to heme and transfer to cysteine has not been worked out: NO bound to the deoxygenated ferrous heme of hemoglobin will not directly transfer to a cysteine without the removal of an electron to form NO^+^ or a thiyl radical (a one-electron oxidation to form the covalent R-SNO). Indeed, no mechanism for this electron transfer step has been identified in the last two decades. From a physiological standpoint, the initial SNO-hemoglobin hypothesis was supported by the measurement of arterial-to-venous gradient that suggested artery-to-vein delivery of NO from Cys93 (132). However, these initial high levels and arterial-to-venous gradient of SNO-hemoglobin detected in vivo have not been reproduced, and the hypoxia-dependent release of NO has been difficult to assess and reproduce (133, 135–137). Perhaps most importantly, a Cys93Ala mutant mouse model was created and studied and appears to have intact physiological hypoxic vasodilation (138). One study suggesting an abnormal hypoxic response in this mouse model did not test canonical hypoxic vasodilation in vivo, only the microcirculatory vasodilatory response to ischemia (flow-mediated vasodilation) (139). A recent study used multiple assays to comprehensively test RBC-mediated NO signaling using red cells from the Cys93Ala mouse and found that signaling effects were evident with or without the Cys93 residue (140). From our analysis of the literature, it appears that SNO-hemoglobin can form under conditions of high NO flux, like sepsis, and when formed can contribute to vasodilation, but a dynamic hypoxia-redox response and a role in physiological NO signaling have not yet been established.

### Nitrite Reductase Hypothesis

A third pathway proposed that links erythrocyte and hemoglobin deoxygenation to NO signaling is the nitrite reductase hypothesis (Figure 2d). This hypothesis posits that a classical electron and proton transfer reaction converts nitrite to NO, catalyzed by electron transfer from the deoxygenated ferrous heme of hemoglobin (137, 141, 142): nitrite (NO_2_^−^) + deoxyhemoglobin (Fe^2+^) + H^+^ − nitric oxide (NO) + methemoglobin (Fe^3+^) + OH^−^.

This hypothesis was advanced based on human translational studies showing that nitrite in plasma and RBCs has arterial-to-venous gradient, suggesting nitrite consumption across the circulation (137) and potent vasodilation during pharmacological nitrite infusions (137, 141). Unlike SNO-hemoglobin, the nitrite arterial-to-venous gradient and vasodilation by nitrite have been reproduced by numerous investigators (e.g., 135). In the presence of deoxygenated red cells in blood and in vitro, nitrite is converted to NO, which can be detected by NO formation in the RBC, where it binds to other deoxygenated hemoglobin molecules to form iron-nitrosyl-heme (Fe+^2^-NO) (141). NO formation from the reaction of nitrite with deoxyhemoglobin or deoxygenated erythrocytes has been shown to activate platelet-soluble guanylate cyclase, increase cGMP levels, inhibit platelet activation (137), and inhibit mitochondrial respiration via NO-dependent inhibition of complex IV (137, 143). The reaction of nitrite with deoxygenated hemoproteins has been broadly confirmed across all ferrous hemoproteins, including myoglobin, Cygb, Ngb, globin X, and nonhemoglobin redox active heme and molybdenum enzymes, suggesting broadly conserved pathways for hypoxic NO signaling via nitrite reduction (recently reviewed in 144).

The chemistry of the reaction of nitrite with hemoglobin has been extensively studied, and there is little controversy about the underlying mechanism. The reaction (shown above) is a second-order reaction, such that the rate of NO formation is dependent on the concentrations of nitrite and deoxygenated heme on hemoglobin (or heme in other globins and enzymes) (142). However, the rate of all second-order reactions also depends on the intrinsic reactivity with the heme, which is characterized by the bimolecular rate constant K. Interestingly, in hemoglobin this rate constant is in fact not constant and changes with the allosteric conformation of the R (oxy)-state and the T (deoxy)-state. The R-state rate constant is about 30 times faster than the T-state. This means that the rate of nitrite reduction and NO formation by hemoglobin is fastest during rapid deoxygenation of hemoglobin, when most of the hemoglobin is in the oxygenated R-state, but O_2_ molecules are released, freeing deoxygenated hemes for nitrite binding and conversion to NO. This rate is maximal around the P_50_ of hemoglobin, where 50% of the hemoglobin is deoxygenated. This chemistry is called R-state catalysis and provides a significant mechanism to link regional O_2_ utilization and O_2_ delivery to NO formation via nitrite reduction (142).

A major challenge for the nitrite reductase hypothesis has been how NO can escape from an RBC and avoid autocapture by vicinal deoxygenated hemoglobin, which has an extremely high NO affinity. One solution is that NO does, in fact, diffuse out from reactions at the erythrocyte membrane in an inefficient process. This is supported by studies that detect NO formation and release from nitrite-treated erythrocytes (137). The other possibility is that NO can be trapped on heme and released from heme, via a process called oxidative denitrosylation (137). NO_2_^·^ and H_2_O_2_ are both intermediates that result from redox reactions between nitrite and oxygenated hemoglobin, which can both oxidize the heme–NO, facilitating the release of NO from ironnitrosyl-hemoglobin by oxidative denitrosylation (137, 145). This reaction also occurs around the P_50_ of hemoglobin (where auto-oxidation and nitrite oxidation are maximal). Finally, the reaction of nitrite and deoxyhemoglobin can generate a higher NO, N_2_O_3_, via reactions of NO with nitrogen dioxide (NO_2_^·^). Formation of N_2_O_3_ can result from radical–radical interactions between NO and NO_2_^·^ and innersphere and outersphere reactions of NO and nitrite with ferric hemoglobin (145). The N_2_O_3_ molecule is more stable in hemoglobin solutions and could diffuse out of the RBC and homolyze to NO and NO_2_ or *S*-nitrosate plasma thiols, both pathways to the activation of sGC and vasodilation. This may explain how RBC eNOS-derived NO exits the RBC to modulate blood pressure and blood flow and limit cardiac ischemic injury, as reported by Wood et al. (146) and Leo et al. (147).

Thus, while the formation of NO and signaling of nitrite and deoxygenated hemoglobin, other globins, and deoxygenated RBCs have been extensively validated, the mediators and mechanisms that allow such signaling in the presence of high concentrations of scavenging globins are areas of active and continued investigation.

## FUTURE DIRECTIONS

This review provides a generalized overview of blood pressure regulation through the interplay between RNS/ROS, redox switches, and Ca^2+^ signaling. Over the past few decades, studies using global antioxidant-focused approaches for fighting aging and diseases associated with oxidative stress (e.g., diabetes, cancer, and neurodegeneration) have largely failed, indicating that the global cellular redox state represents a poor therapeutic target. Meng et al. introduced the 5R principles of a precision redox approach: “Right species, Right place, Right time, Right level and Right target” (4, p. 1069). These principles focus on roles of redox switches in cellular compartments and confined spaces and the association of common gene polymorphisms to cardiovascular diseases. Importantly, the relative differences in redox signaling and reliance on Ca^2+^, NO, or EDHF as stimuli for vascular relaxation in large versus small vessels, detailed in this review, highlight the importance of distinguishing local from global ROS for improved understanding of cardiovascular pathologies.

The precise regulation of vascular tone is a chain of complex reactions involving signal transduction between different cell types to adapt to neurohumoral or mechanical stimuli. These pathways involve numerous enzymes regulated by signaling molecules (i.e.,ROS/RNS,K^+^,Ca^2+^), for which directional changes in activity may be detrimental to disease progression. Identifying the precise interplay between oxidants and reductants regulating Ca^2+^ signaling pathways or vice versa represents an interesting new direction in vascular biology. The recent availability of technologies that allow preclinical study of oxidation-reduction events at the subcellular level, such as the organelle or interorganellar interface with targeted GSH- and H_2_O_2_-sensing probes, may help with understanding the spatiotemporal redox events associated with cellular Ca^2+^ transients in ECs and VSMCs (148).

The key redox regulated players and pathways in EC and VSMC Ca^2+^-dependent vasoregulation were discussed in the first three sections of this review. Members of the voltage- and nonvoltage-gated channels and their regulators were shown to be under the direct or indirect regulation of redox switches. Nonetheless, there are other as-yet-uncharacterized targets and modulators. As an example, CYB5R3 was shown to regulate heme redox state in both ECs and VSMCs; however, whether it has a role in regulating other nonheme targets remains unstudied. There are four other members of the CYB5R family expressed in the vasculature, but their cellular localization and role in cellular processes remain undetermined. It is possible that different CYB5R isoforms may impact numerous Ca^2+^ channels and their regulators in vascular cells.

The study of common genetic variants impacting redox signaling and switches in cardiovascular disease and the functional consequences caused by these variants is also an emerging field. For example, the common polymorphism in SOD2 (SOD2_V16A_) in patients with sickle cell disease associates with increased cardiovascular consequences by curtailing mitochondrial complex IV activity and increasing ROS production (149, 150). Further identification of additional genetic biomarkers may lead to better precision medicine approaches for predicting the right therapy for the right patient.
